# Idioms in the World: A Focus on Processing

**DOI:** 10.3389/fpsyg.2019.01155

**Published:** 2019-05-24

**Authors:** Elena S. Kulkova, Martin H. Fischer

**Affiliations:** Department of Psychology, University of Potsdam, Potsdam, Germany

**Keywords:** embodiment, figurative language, metaphor, idiom, processing

Recent experiment-based psycho- and neuro-linguistic research brought new insights into language processing mechanisms and meaning representation in the brain. More specifically, it highlighted the dynamic nature of brain connections and a constant interplay between distributed neuronal circuits during meaning processing. These developments led to a shift from an amodal view, which perceives conceptual information activation as parallel to and independent from adjoining neural activation in sensorimotor circuits (Mahon and Caramazza, 2008; Meteyard et al., 2012), to the embodied cognition view that highlights the role of sensorimotor experience in the formation of flexible, distributed conceptual representations encompassing features acquired via different perceptual modalities (Fischer and Zwaan, 2008; Barsalou, 2010). Embodied cognition, therefore, suggests that conceptual knowledge and, consequently, semantic knowledge are grounded in bodily experience and situated actions (Glenberg et al., 2008; Pulvermüller, 2013). However, currently there is a tendency toward perceiving embodied and disembodied views not as mutually exclusive distinct theories, but as bridging a gap between them. The hub and spoke model and the sensory-motor model demonstrate attempts to integrate the amodal and modality-specific views (see Mahon, 2015).

A broad range of behavioral, physiological, and neuroimaging data demonstrating co-activation of language- and action-related brain areas support this claim with regard to concrete language (Binder et al., 2005; Pulvermüller et al., 2005; Barsalou, 2008; Hauk et al., 2008). However, the data are less conclusive with regard to figurative expressions, which constitute a significant part of language. One of the reasons is that figurative language subsumes a wide variety of heterogeneous phenomena (metonymy, idioms, metaphors, proverbs, hyperbole, irony) which differ syntactically (from phrasal verbs to compounds and even sentences), as well as in their properties (familiarity, ambiguity, transparency, compositionality, salience, predictability) and essential features (although both irony and hyperbole are based on cognitive contrast, it is a contrast *of kind* for irony and a contrast *in magnitude* for hyperbole; Hsiao and Lily, 2010). This diversity and complexity of non-literal language types does not allow for clear-cut and strictly defined boundaries; it has led to a distinction of non-literal phenomena not dichotomously, but along a conventionality continuum (Cacciari and Papagno, 2012).

Secondly, the linguistic phenomena, embraced by the broad term “non-literal language” have been analyzed to different degrees of detail. Specifically, different aspects of metaphor production comprehension and use have been extensively studied (Gibbs, 2008, 2015; Schmidt and Seger, 2009; Bambini et al., 2011; Gibbs and Colston, 2012; Forgács et al., 2014; Obert et al., 2014; Lai and Desai, 2016; Briner et al., 2018; Rataj et al., 2018; Reilly et al., 2019). Furthermore, metaphors represent a powerful cognitive device guided by environmental experiences, which enabled the studies of metaphor framing influences not only on linguistic communication *per se*, but also on judgments, reasoning, intentions, and actions (Robins and Mayer, 2000; Slepian et al., 2010; Thibodeau and Boroditsky, 2011, 2013; Landau et al., 2014; Marin et al., 2014; Hauser and Schwarz, 2015; Elmore and Luna-Lucero, 2017; Thibodeau et al., 2017). Despite considerable research (Gibbs and Nayak, 1989; Cacciari and Tabossi, 1993; Mashal et al., 2008; Vulchanova et al., 2011; Cuccio et al., 2014; Häuser et al., 2016; Cacciari et al., 2018), the study of idioms still leaves open for debate the questions of defining idioms or differentiating them from other types of non-literal expressions (Cacciari, 2014). One of the main confusions is in defining idioms from metaphors, as it was debated whether idiom processing is possible without constant recourse to conceptual metaphors (Owens, 2016). However, although some idioms are indeed derived from metaphors and can still be partially motivated by conceptual mappings between domains (Gibbs, 1992), idioms as a class comprising syntactically and compositionally differing phenomena (Caillies and Butcher, 2007) are divergent from metaphors. The crucial difference is that idiomatic meaning is predominantly fixed and conventional, and it can be modified but not changed when used in various contexts. Metaphoric meaning, in turn, is flexible and intricate, can be profoundly changed by the context, and therefore always requires online construction (Cacciari, 2014; Bambini et al., 2016). Distinctive neural correlates for processing of idioms (left MTG and left IFG, involved in selection-inhibition operations) and metaphors [left precentral gyrus (BA 6), linking concrete and abstract domains and the left inferior parietal lobe (IPL), executing higher-order cognitive motor functions; Fogassi and Luppino, 2005] also argue against conflating them.

Disregarding these principal differences between the two linguistic forms results in their interchangeable use (e.g., Aziz-Zadeh et al., 2006), which, in turn, may posit serious confoundment, as comprehending these figurative devices that have different mental representations engages dissimilar cognitive mechanisms; based on both semantic and structural analysis of meaning and retrieval from semantic memory during idiom processing, and focused on the conceptual models and templates underlying metaphor meaning construction. Vulchanova et al. (2019) provide a detailed overview of the models of figurative language processing.

Recent studies on processing non-literal expressions with action-related semantics reported activation of motoric brain areas during either literal (Raposo et al., 2009), metaphoric (Desai et al., 2011), or only during metaphor but not idiom processing (Cacciari et al., 2011; Desai et al., 2013). Only limited publications present evidence of sensorimotor engagement during idiomatic meaning comprehension (Boulenger et al., 2009, 2012). Overall, the studies emphasize the role of context in meaning disambiguation and suggest that an increase in abstractness of the language stimuli leads to a decrease in the sensorimotor system's involvement (especially in case of idioms). However, these results could be interpreted not in favor of idiom disembodiment, but as a demonstration of different processing schemas that idioms and metaphors employ: the dual-reference idiomatic nature enables engagement of a hybrid processing mechanism that encompasses both compositional and holistic context-based analysis during idiom comprehension (Caillies and Butcher, 2007; Boulenger et al., 2012; Cacciari and Pesciarelli, 2013). Metaphors, which retain stronger links to the original meaning of the constituent words, rely more on online mental simulation to compute complex, flexible meanings.

Engaging different processing mechanisms may result in spatially and temporally different patterns of neurocognitive involvement (Rapp et al., 2012; Yang and Shu, 2016). For example, Cacciari et al. (2011) reported no motor engagement in idiom processing, but single-pulse TMS applied at the end of sentences to register meaning-induced MEPs could be inefficient to record idiom-induced motor activation, since idioms are processed online (mentally simulated) only until the idiom is recognized, and then a switch to the non-compositional mode (retrieval from semantic memory) occurs. Lack of motor engagement during idiom comprehension can be explained by heterogeneity of idioms: e.g., Raposo et al. (2009) used highly familiar and opaque idioms, which minimized the need for mental simulation during their processing and consequently may have reduced the level of sensorimotor cortical activation. Therefore, idiomatic meaning may be less embodied compared to metaphoric meaning, but not totally disembodied.

This evidence highlights the need for a more profound exploration of properties specific to figurative language types and subtypes of each phenomenon, which could considerably benefit present-stage figurative language research and promote a better understanding of the mechanisms the human brain employs for their acquisition, production, and processing. This will provide an integrative theoretical model that can more comprehensively and consistently outline the cognitive mechanisms and neural circuitry underlying processing of heterogeneous and multifaceted figurative language. Taken together, it will inform the development of more precise neuro-cognitive models, support AI applications and enhance understanding of language processing in general.

## Author Contributions

EK and MF have contributed equally to this submission.

### Conflict of Interest Statement

The authors declare that the research was conducted in the absence of any commercial or financial relationships that could be construed as a potential conflict of interest.
